# Supplementary material for: Multi‐dataset identification of innovative feature genes and molecular mechanisms in keratoconus
Source: J Cell Mol Med. 2024 Sep 19;28(18):e70079. doi: 10.1111/jcmm.70079 (PMC11412914; doi:10.1111/jcmm.70079)
Supplement: Supplementary file 2 — Material S2. [file JCMM-28-e70079-s002.zip › 7.GSEA analysis/3.DBNDD1/KEGG_ABC_TRANSPORTERS.html]

Details for gene set KEGG\_ABC\_TRANSPORTERS[GSEA]

|  || Dataset | DBNDD1.group.cls#h\_versus\_l |
| Phenotype | group.cls#h\_versus\_l |
| Upregulated in class | h |
| GeneSet | KEGG\_ABC\_TRANSPORTERS |
| Enrichment Score (ES) | 0.28018522 |
| Normalized Enrichment Score (NES) | 0.8425945 |
| Nominal p-value | 0.6543967 |
| FDR q-value | 1.0 |
| FWER p-Value | 1.0 |
Table: GSEA Results Summary

  

Fig 1: Enrichment plot: KEGG\_ABC\_TRANSPORTERS      
 Profile of the Running ES Score & Positions of GeneSet Members on the Rank Ordered List

  

| SYMBOL | TITLE | RANK IN GENE LIST | RANK METRIC SCORE | RUNNING ES | CORE ENRICHMENT || 1 | ABCC8 | na | 1099 | 0.372 | 0.0353 | Yes |
| 2 | ABCB5 | na | 1554 | 0.332 | 0.0818 | Yes |
| 3 | ABCA3 | na | 2273 | 0.283 | 0.1120 | Yes |
| 4 | ABCA4 | na | 3318 | 0.239 | 0.1248 | Yes |
| 5 | ABCG5 | na | 3513 | 0.231 | 0.1608 | Yes |
| 6 | ABCA10 | na | 4107 | 0.210 | 0.1815 | Yes |
| 7 | ABCB1 | na | 4150 | 0.209 | 0.2178 | Yes |
| 8 | ABCA8 | na | 4243 | 0.205 | 0.2520 | Yes |
| 9 | ABCD1 | na | 5834 | 0.155 | 0.2340 | Yes |
| 10 | ABCB4 | na | 6004 | 0.150 | 0.2562 | Yes |
| 11 | ABCC9 | na | 6500 | 0.137 | 0.2665 | Yes |
| 12 | ABCD4 | na | 7361 | 0.117 | 0.2627 | Yes |
| 13 | ABCA2 | na | 7468 | 0.114 | 0.2802 | Yes |
| 14 | ABCA9 | na | 8473 | 0.095 | 0.2683 | No |
| 15 | ABCC2 | na | 9058 | 0.085 | 0.2667 | No |
| 16 | ABCA1 | na | 9231 | 0.082 | 0.2765 | No |
| 17 | ABCB8 | na | 9990 | 0.071 | 0.2675 | No |
| 18 | ABCB10 | na | 12615 | 0.042 | 0.1994 | No |
| 19 | ABCA7 | na | 16170 | 0.015 | 0.0995 | No |
| 20 | ABCC10 | na | 16326 | 0.014 | 0.0975 | No |
| 21 | ABCC5 | na | 16745 | 0.011 | 0.0875 | No |
| 22 | ABCA5 | na | 17381 | 0.007 | 0.0704 | No |
| 23 | ABCC12 | na | 17540 | 0.006 | 0.0669 | No |
| 24 | ABCA12 | na | 18416 | -0.000 | 0.0418 | No |
| 25 | ABCG1 | na | 18471 | -0.001 | 0.0403 | No |
| 26 | ABCC6 | na | 19021 | -0.005 | 0.0253 | No |
| 27 | ABCB7 | na | 20296 | -0.016 | -0.0086 | No |
| 28 | TAP1 | na | 20842 | -0.021 | -0.0206 | No |
| 29 | TAP2 | na | 21858 | -0.031 | -0.0444 | No |
| 30 | ABCB9 | na | 22229 | -0.034 | -0.0489 | No |
| 31 | ABCC11 | na | 22570 | -0.038 | -0.0518 | No |
| 32 | ABCD3 | na | 22653 | -0.039 | -0.0472 | No |
| 33 | ABCC4 | na | 22663 | -0.039 | -0.0404 | No |
| 34 | ABCB6 | na | 22889 | -0.042 | -0.0394 | No |
| 35 | ABCC1 | na | 25913 | -0.086 | -0.1111 | No |
| 36 | CFTR | na | 27609 | -0.121 | -0.1382 | No |
| 37 | ABCC3 | na | 28896 | -0.154 | -0.1476 | No |
| 38 | ABCA13 | na | 29320 | -0.167 | -0.1298 | No |
| 39 | ABCD2 | na | 29490 | -0.172 | -0.1038 | No |
| 40 | ABCG8 | na | 30988 | -0.220 | -0.1073 | No |
| 41 | ABCA6 | na | 31266 | -0.231 | -0.0737 | No |
| 42 | ABCG2 | na | 32696 | -0.306 | -0.0598 | No |
| 43 | ABCB11 | na | 32804 | -0.314 | -0.0064 | No |
| 44 | ABCG4 | na | 33193 | -0.344 | 0.0443 | No |
Table: GSEA details [plain text format]

  

Fig 2: KEGG\_ABC\_TRANSPORTERS      
 Blue-Pink O' Gram in the Space of the Analyzed GeneSet

  

Fig 3: KEGG\_ABC\_TRANSPORTERS: Random ES distribution      
 Gene set null distribution of ES for **KEGG\_ABC\_TRANSPORTERS**
